# Supplementary material for: Assessing equity in the uptake of remote foot temperature monitoring in a large integrated US healthcare system
Source: PLoS One. 2024 Apr 1;19(4):e0301260. doi: 10.1371/journal.pone.0301260 (PMC10984545; doi:10.1371/journal.pone.0301260)
Supplement: S1 Table — (PDF) [file pone.0301260.s001.pdf]

S1 Table. International Classification of Diseases, 10th edition (ICD-10) codes for ulceration

TYPE	Code	ICDDescription
ICD DX	E08.621	DIABETES MELLITUS DUE TO UNDERLYING CONDITION WITH FOOT ULCER
ICD DX	E09.621	DRUG OR CHEMICAL INDUCED DIABETES MELLITUS WITH FOOT ULCER
ICD DX	E10.621	TYPE 1 DIABETES MELLITUS WITH FOOT ULCER
ICD DX	E11.621	TYPE 2 DIABETES MELLITUS WITH FOOT ULCER
ICD DX	E13.621	OTHER SPECIFIED DIABETES MELLITUS WITH FOOT ULCER
ICD DX	I70.234	ATHEROSCLEROSIS OF NATIVE ARTERIES OF RIGHT LEG WITH ULCERATION OF HEEL AND MIDFOOT
ICD DX	I70.235	ATHEROSCLEROSIS OF NATIVE ARTERIES OF RIGHT LEG WITH ULCERATION OF OTHER PART OF FOOT
ICD DX	I70.244	ATHEROSCLEROSIS OF NATIVE ARTERIES OF LEFT LEG WITH ULCERATION OF HEEL AND MIDFOOT
ICD DX	I70.245	ATHEROSCLEROSIS OF NATIVE ARTERIES OF LEFT LEG WITH ULCERATION OF OTHER PART OF FOOT
ICD DX	I70.334	ATHEROSCLEROSIS OF UNSPECIFIED TYPE OF BYPASS GRAFT(S) OF THE RIGHT LEG WITH ULCERATION OF HEEL AND MIDFOOT
ICD DX	I70.335	ATHEROSCLEROSIS OF UNSPECIFIED TYPE OF BYPASS GRAFT(S) OF THE RIGHT LEG WITH ULCERATION OF OTHER PART OF FOOT
ICD DX	I70.344	ATHEROSCLEROSIS OF UNSPECIFIED TYPE OF BYPASS GRAFT(S) OF THE LEFT LEG WITH ULCERATION OF HEEL AND MIDFOOT
ICD DX	I70.345	ATHEROSCLEROSIS OF UNSPECIFIED TYPE OF BYPASS GRAFT(S) OF THE LEFT LEG WITH ULCERATION OF OTHER PART OF FOOT
ICD DX	I70.434	ATHEROSCLEROSIS OF AUTOLOGOUS VEIN BYPASS GRAFT(S) OF THE RIGHT LEG WITH ULCERATION OF HEEL AND MIDFOOT
ICD DX	I70.435	ATHEROSCLEROSIS OF AUTOLOGOUS VEIN BYPASS GRAFT(S) OF THE RIGHT LEG WITH ULCERATION OF OTHER PART OF FOOT
ICD DX	I70.444	ATHEROSCLEROSIS OF AUTOLOGOUS VEIN BYPASS GRAFT(S) OF THE LEFT LEG WITH ULCERATION OF HEEL AND MIDFOOT
ICD DX	I70.445	ATHEROSCLEROSIS OF AUTOLOGOUS VEIN BYPASS GRAFT(S) OF THE LEFT LEG WITH ULCERATION OF OTHER PART OF FOOT
ICD DX	I70.534	ATHEROSCLEROSIS OF NONAUTOLOGOUS BIOLOGICAL BYPASS GRAFT(S) OF THE RIGHT LEG WITH ULCERATION OF HEEL AND MIDFOOT
ICD DX	I70.535	ATHEROSCLEROSIS OF NONAUTOLOGOUS BIOLOGICAL BYPASS GRAFT(S) OF THE RIGHT LEG WITH ULCERATION OF OTHER PART OF FOOT
ICD DX	I70.544	ATHEROSCLEROSIS OF NONAUTOLOGOUS BIOLOGICAL BYPASS GRAFT(S) OF THE LEFT LEG WITH ULCERATION OF HEEL AND MIDFOOT
ICD DX	I70.545	ATHEROSCLEROSIS OF NONAUTOLOGOUS BIOLOGICAL BYPASS GRAFT(S) OF THE LEFT LEG WITH ULCERATION OF OTHER PART OF FOOT
ICD DX	I70.634	ATHEROSCLEROSIS OF NONBIOLOGICAL BYPASS GRAFT(S) OF THE RIGHT LEG WITH ULCERATION OF HEEL AND MIDFOOT

TYPE	Code	ICDDescription
ICD DX	I70.635	ATHEROSCLEROSIS OF NONBIOLOGICAL BYPASS GRAFT(S) OF THE RIGHT LEG WITH ULCERATION OF OTHER PART OF FOOT
ICD DX	I70.734	ATHEROSCLEROSIS OF OTHER TYPE OF BYPASS GRAFT(S) OF THE RIGHT LEG WITH ULCERATION OF HEEL AND MIDFOOT
ICD DX	I70.735	ATHEROSCLEROSIS OF OTHER TYPE OF BYPASS GRAFT(S) OF THE RIGHT LEG WITH ULCERATION OF OTHER PART OF FOOT
ICD DX	L97.401	NON-PRESSURE CHRONIC ULCER OF UNSPECIFIED HEEL AND MIDFOOT LIMITED TO BREAKDOWN OF SKIN
ICD DX	L97.402	NON-PRESSURE CHRONIC ULCER OF UNSPECIFIED HEEL AND MIDFOOT WITH FAT LAYER EXPOSED
ICD DX	L97.403	NON-PRESSURE CHRONIC ULCER OF UNSPECIFIED HEEL AND MIDFOOT WITH NECROSIS OF MUSCLE
ICD DX	L97.404	NON-PRESSURE CHRONIC ULCER OF UNSPECIFIED HEEL AND MIDFOOT WITH NECROSIS OF BONE
ICD DX	L97.409	NON-PRESSURE CHRONIC ULCER OF UNSPECIFIED HEEL AND MIDFOOT WITH UNSPECIFIED SEVERITY
ICD DX	L97.411	NON-PRESSURE CHRONIC ULCER OF RIGHT HEEL AND MIDFOOT LIMITED TO BREAKDOWN OF SKIN
ICD DX	L97.412	NON-PRESSURE CHRONIC ULCER OF RIGHT HEEL AND MIDFOOT WITH FAT LAYER EXPOSED
ICD DX	L97.413	NON-PRESSURE CHRONIC ULCER OF RIGHT HEEL AND MIDFOOT WITH NECROSIS OF MUSCLE
ICD DX	L97.414	NON-PRESSURE CHRONIC ULCER OF RIGHT HEEL AND MIDFOOT WITH NECROSIS OF BONE
ICD DX	L97.419	NON-PRESSURE CHRONIC ULCER OF RIGHT HEEL AND MIDFOOT WITH UNSPECIFIED SEVERITY
ICD DX	L97.421	NON-PRESSURE CHRONIC ULCER OF LEFT HEEL AND MIDFOOT LIMITED TO BREAKDOWN OF SKIN
ICD DX	L97.422	NON-PRESSURE CHRONIC ULCER OF LEFT HEEL AND MIDFOOT WITH FAT LAYER EXPOSED
ICD DX	L97.423	NON-PRESSURE CHRONIC ULCER OF LEFT HEEL AND MIDFOOT WITH NECROSIS OF MUSCLE
ICD DX	L97.424	NON-PRESSURE CHRONIC ULCER OF LEFT HEEL AND MIDFOOT WITH NECROSIS OF BONE
ICD DX	L97.429	NON-PRESSURE CHRONIC ULCER OF LEFT HEEL AND MIDFOOT WITH UNSPECIFIED SEVERITY
ICD DX	L97.501	NON-PRESSURE CHRONIC ULCER OF OTHER PART OF UNSPECIFIED FOOT LIMITED TO BREAKDOWN OF SKIN
ICD DX	L97.502	NON-PRESSURE CHRONIC ULCER OF OTHER PART OF UNSPECIFIED FOOT WITH FAT LAYER EXPOSED
ICD DX	L97.503	NON-PRESSURE CHRONIC ULCER OF OTHER PART OF UNSPECIFIED FOOT WITH NECROSIS OF MUSCLE
ICD DX	L97.504	NON-PRESSURE CHRONIC ULCER OF OTHER PART OF UNSPECIFIED FOOT WITH NECROSIS OF BONE
ICD DX	L97.509	NON-PRESSURE CHRONIC ULCER OF OTHER PART OF UNSPECIFIED FOOT WITH UNSPECIFIED SEVERITY
ICD DX	L97.511	NON-PRESSURE CHRONIC ULCER OF OTHER PART OF RIGHT FOOT LIMITED TO BREAKDOWN OF SKIN
ICD DX	L97.512	NON-PRESSURE CHRONIC ULCER OF OTHER PART OF RIGHT FOOT WITH FAT LAYER EXPOSED

TYPE	Code	ICDDescription
ICD DX	L97.513	NON-PRESSURE CHRONIC ULCER OF OTHER PART OF RIGHT FOOT WITH NECROSIS OF MUSCLE
ICD DX	L97.514	NON-PRESSURE CHRONIC ULCER OF OTHER PART OF RIGHT FOOT WITH NECROSIS OF BONE
ICD DX	L97.519	NON-PRESSURE CHRONIC ULCER OF OTHER PART OF RIGHT FOOT WITH UNSPECIFIED SEVERITY
ICD DX	L97.521	NON-PRESSURE CHRONIC ULCER OF OTHER PART OF LEFT FOOT LIMITED TO BREAKDOWN OF SKIN
ICD DX	L97.522	NON-PRESSURE CHRONIC ULCER OF OTHER PART OF LEFT FOOT WITH FAT LAYER EXPOSED
ICD DX	L97.523	NON-PRESSURE CHRONIC ULCER OF OTHER PART OF LEFT FOOT WITH NECROSIS OF MUSCLE
ICD DX	L97.524	NON-PRESSURE CHRONIC ULCER OF OTHER PART OF LEFT FOOT WITH NECROSIS OF BONE
ICD DX	L97.529	NON-PRESSURE CHRONIC ULCER OF OTHER PART OF LEFT FOOT WITH UNSPECIFIED SEVERITY
